# Antagonizing Retinoic Acid Receptors Increases Myeloid Cell Production by Cultured Human Hematopoietic Stem Cells

**DOI:** 10.1007/s00005-016-0411-0

**Published:** 2016-07-13

**Authors:** Geoffrey Brown, Aleksandra Marchwicka, Alan Cunningham, Kai-Michael Toellner, Ewa Marcinkowska

**Affiliations:** 1 Institute of Clinical Sciences, College of Medical and Dental Sciences, University of Birmingham, Edgbaston, Birmingham, B15 2TT UK; 2 Institute of Immunology and Immunotherapy, College of Medical and Dental Sciences, University of Birmingham, Edgbaston, Birmingham, B15 2TT UK; 3 Laboratory of Protein Biochemistry, Faculty of Biotechnology, University of Wroclaw, Wroclaw, Poland

**Keywords:** Retinoic acid receptor, Hematopoiesis, Neutrophils, Monocytes, All-*trans* retinoic acid, Agonist, Antagonist

## Abstract

Activities of the retinoic acid receptor (RAR)α and RARγ are important to hematopoiesis. Here, we have investigated the effects of receptor selective agonists and antagonists on the primitive human hematopoietic cell lines KG1 and NB-4 and purified normal human hematopoietic stem cells (HSCs). Agonizing RARα (by AGN195183) was effective in driving neutrophil differentiation of NB-4 cells and this agonist synergized with a low amount (10 nM) of 1α,25-dihydroxyvitamin D_3_ to drive monocyte differentiation of NB-4 and KG1 cells. Treatment of cultures of human HSCs (supplemented with stem cell factor ± interleukin 3) with an antagonist of all RARs (AGN194310) or of RARα (AGN196996) prolonged the lifespan of cultures, up to 55 days, and increased the production of neutrophils and monocytes. Slowing down of cell differentiation was not observed, and instead, hematopoietic stem and progenitor cells had expanded in number. Antagonism of RARγ (by AGN205728) did not affect cultures of HSCs. Studies of CV-1 and LNCaP cells transfected with RAR expression vectors and a reporter vector revealed that RARγ and RARβ are activated by sub-nM all-*trans* retinoic acid (EC_50_–0.3 nM): ~50-fold more is required for activation of RARα (EC_50_–16 nM). These findings further support the notion that the balance of expression and activity of RARα and RARγ are important to hematopoietic stem and progenitor cell expansion and differentiation.

## Introduction

Retinoic acid receptors (RARs) are members of the nuclear hormone receptor superfamily, and there are three main isoforms of RAR in vertebrates: RARα, β, and γ (Chambon 1996; Sucov and Evans 1995). RARs form heterodimers with retinoid X receptors which bind to retinoic acid response elements (RAREs) in the promoter/enhancer regions of target genes to either activate or repress gene transcription (Kastner et al. 1997). Activation versus repression of transcription by RARs is affected by binding or otherwise of the natural ligand all-*trans* retinoic acid (ATRA) which influences the recruitment of either corepressors or coactivators of transcription (Niederreither and Dollé 2008). In the absence of ATRA, RARα binds the silencing mediator of retinoic acid and thyroid hormone receptor/nuclear receptor corepressor family of corepressors resulting in the formation of a histone deacetylase repressor complex at RAREs and repression of transcription. Binding of ATRA to RARα leads to the release of corepressors, recruitment of coactivators, and gene transcription. In contrast to RARα, β and γ have been reported to activate gene transcription without having bound ligand, and in this case, binding of ATRA serves to increase activation (Farboud et al. 2003; Hauksdottir et al. 2003).

RARs are important regulators of vertebrate development as to cells making fate decisions and then undergoing differentiation (reviewed in Mendoza-Parra and Gronemeyer 2013). Expression of the different isoforms varies temporally during development and, in regard to cell type, pointing to the isoforms having different functions rather than functional redundancy (Dollé et al. 1990; Germain et al. 2006; Kastner et al. 1995). Findings from RAR-knockout mice emphasize the importance of RARs to development. Ocular defects and reduced body weight are seen in RARβ-knockout mice, RARβ-knockout mice have severe defects, and knockout of two or more receptors is generally lethal (Ghyselinck et al. 1997; Li et al. 1993; Lohnes et al. 1993; Subbarayan et al. 1997). There are not obvious defects in the RARα-knockout mouse, and in humans, abnormality in regard to expression/function of this isoform is associated with malignancy. In acute promyelocytic leukemia (APL), chromosome translocations lead to chimeric RARα proteins that result in a block in myeloid cell differentiation at the promyelocyte stage (reviewed in Ablain and de Thé 2014). As to other isoforms and malignancy, RARγ is reported to be an oncogene in hepatocellular carcinoma (Yan et al. 2010).

RARα and RARγ are important regulators of the differentiation of hematopoietic cells. Agonizing RARα, using ATRA or a selective agonist, promotes the differentiation of normal myeloid progenitor cells (Gratas et al. 1993) and promyeloid cell lines, such as HL60 cells, which respond by differentiating towards neutrophils (Breitman et al. 1980). ATRA may also be involved in specifying a granulocyte fate, as this agent appears to orient pluripotent hematopoietic progenitors towards the granulocyte lineage (Tocci et al. 1996). In keeping with these roles for RARα, the RAR fusion proteins that arrest myeloid differentiation of APL cells function as dominant-negative inhibitors of wild-type RARα (reviewed in Tsai and Collins 1993; Yan et al. 2010). A shift provoked by the fusion proteins to attract a novel repertoire of corepressors has been proposed to contribute to this action (Mengeling et al. 2011). Though ATRA clearly promotes neutrophil differentiation, the influence of RARα is modulatory: RARα is dispensable as evidenced by RARα^−/−^ mice which make neutrophils. Kastner concluded that RARα modulates granulopoiesis in a bi-directional manner, with ligand-bound receptor promoting differentiation and ligand-free receptor inhibiting it (Kastner et al. 2001).

Agonizing RARγ appears to oppose the ligand-driven action of RARα by interfering with the capacity of hematopoietic stem cells (HSCs) to undergo differentiation and promoting self-renewal and/or proliferation. A reduced number of HSCs in the γ-knockout mouse highlight the importance of RARγ to hematopoiesis, and loss of RARγ also abrogated the capacity of ATRA to potentiate the maintenance of HSC in culture. Purton et al. (2006) concluded that RARγ plays a critical role in regulating whether HSC self-renew and maintain their pluripotency versus embark on differentiation. Like RARα, the role of RARγ is modulatory, as HSCs are still present in the knockout mouse. That RARγ has a role in allowing cells to maintain pluripotency, is further supported by the finding that addition of RARγ to the Yamanaka cocktail of transcription factors used to generate induced pluripotent stem cells from somatic cells improves the efficiency, by which these cells can be generated (Wang et al. 2011).

Here, we utilized agonists and antagonists of RARs that are highly selective for RAR receptor isoforms to examine further the influence of RARα and RARγ on the growth and differentiation of promyeloid cell lines and normal human HSCs. In regard to the reported constitutive activity of RARγ, we examine whether transactivation of RARα and RARγ is differentially regulated by ATRA as to concentration dependence.

## Materials and Methods

### Chemicals and Antibodies

1α,25-Dihydroxyvitamin D_3_ (1,25D) was obtained from Cayman Europe (Tallinn, Estonia), while ATRA and TTNPB were from Sigma (St Louis, MO, USA). The compounds were dissolved in absolute ethanol at a concentration of 10 µM and stored at −20 °C. The synthetic retinoids AGN195183, AGN194310, AGN196996, and AGN205728 were synthesized at the Shanghai Institute of Materia Medica. Their synthesis, development, and specificities have been described previously (Hughes et al. 2006; Johnson et al. 1995; Klein et al. 1996; Nagpal et al. 1995; Nagpal and Chandraratna 1996, 2000; Teng et al. 1996). Retinoids were dissolved in 50 % methanol/50 % dimethylsulphoxide (DMSO) at a concentration of 10 mM (stored at −20 °C), and this stock was diluted using culture medium to the required concentration. Rabbit polyclonal antibodies to RARα (sc-550), RARβ (sc-552), and to actin (sc-1616), and a mouse monoclonal antibody to HDAC1 (sc-7872) were from Santa Cruz Biotechnology Inc.

### Cell Lines

NB-4 and KG1 cells were obtained from the German Resource Center for Biological Material (DSMZ GmbH, Braunschweig, Germany). The cells were grown in RPMI-1640 medium supplemented with 10 % fetal bovine serum (FBS), 100 U/ml penicillin, and 100 µg/ml streptomycin (Sigma, St Louis, MO, USA). Cells were grown at 37 °C in 5 % CO_2_. Cell differentiation of NB-4 and KG1 cells was induced by incubating with 10 nM 1,25D ± 1 µM ATRA or 100 nM retinoids for 96 h.

### Primary Cultures of Human CD34^+ve^ Cells

The cells used were CD34^+ve^ cells purified from the blood of normal human adult donors and post-mobilization of stem cells to the blood. Cells obtained and purified in this manner are used routinely by the National Blood Service Stem Cell Laboratory in Birmingham for bone marrow transplantation. Ethics approval for the use of adult human blood-mobilized stem cells (CD34^+ve^huHSC) was from the West Midlands Research Ethics Committee. Informed consent was obtained by the regional National Blood Service Stem Cell Laboratory in Birmingham. CD34^+ve^ cells were purified to 99 % CD34^+ve^, 97 % CD133^+ve^, >99 % lineage^−ve^ (*n* = 15) using an anti-CD34 monoclonal antibody, immunomagnetic beads, and a CliniMACS magnetic separator. Purified cells were plated at 5 × 10^5^ cells/ml in microtiter wells in 200 μl of either RPMI-1640 medium (R and D Systems, Abingdon, UK) or HPGM^TM^ hematopoietic growth medium (Lonza, UK) containing either 10 % FBS or 10 % human serum, antibiotics (100 U/ml penicillin and 100 μg/ml streptomycin) and stem cell factor (SCF), interleukin (IL)-3, and granulocyte-colony stimulating factor (G-CSF) (all from R and D Systems Abingdon, UK) as stated in the results. Cultures were fed every 2–3 days (with fresh compound), split into microtiter wells, and then expanded into 2 ml Costar wells to maintain a cell density of between 2.5 and 10 × 10^5^ cells/ml. Cells were grown at 37 °C in 5 % CO_2_. Viable cells were enumerated by phase contrast microscopy (400× magnification).

### Assays for Progenitor Cells

Assays for week-five cobblestone area forming cells (CAFC), long-term culture-initiating cells (LTC-IC), and colony-forming units (CFU) were performed at the Stem Cell Laboratory at the National Blood Service Stem Cell Laboratory using standard procedures and methods, as described by other workers (de Wynter and Ploemacher 2001; Denning-Kendall et al. 1996; Nicol et al. 1996). CFU assays were performed using Methocult^TM^ GF H8444 (StemCell Technologies) and in 35 mm petri dishes. The presence of CAFC was scored as the presence of 12 or more closely associated and small cells that were embedded in the MS5 cell feeder layer and blind by an experienced reader. The nature of CFUs, as to colony-forming unit-granulocyte/erythroid/megakaryocyte/monocyte (CFU-GEMM), colony-forming unit-granulocyte/macrophage (CFU-GM), burst-forming unit-erythroid (BFU-E), and colony-forming unit-erythroid (CFU-E), was scored blind at the service by a person who routinely reads colony assays and is accredited. The nature of colonies was confirmed by sampling colonies and staining cytocentrifuged preparations of cells.

### Assessment of Cell Differentiation

The expression of differentiation markers at the cell surface was determined by flow cytometry. Differentiation of NB-4 and KG1 cells was measured by staining using the monoclonal antibodies CD11b-FITC and CD14-PE and appropriately labeled isotype controls (all from ImmunoTools, Friesoythe, Germany). Differentiation of human CD34^+ve^ cells was measured by triple-color staining using various cocktails of phycoerythrin-, fluorescein isothyocyanate-, and PerCP-monoclonal antibodies (from Becton–Dickinson, San Jose, CA, USA). The markers used to identify cell populations were as follows: CD45^+ve^ leukocytes; CD34^+ve^/CD133^+ve^ HSCs; CD33^+ve^/CD13^+ve^ immature myeloid; CD117^+ve^ myeloblasts and promyelocytes; CD11b^+ve^/CD15^+ve^ mature myeloid; CD11b^+ve^/CD65^+ve^ neutrophils; CD11b^+ve^/CD14^+ve^ monocytes; glycophorin^+ve^ A erythroid cells; CD61^+ve^ megakaryoblasts and platelets; CD2^+ve^/CD5^+ve^/CD7^+ve^ T cells and NK cells; CD56^+ve^ NK cells; CD19^+ve^/CD20^+ve^ B cells; and CD10^+ve^ B lymphocyte progenitors; and present on mature neutrophils and DR^+ve^ progenitors and present on mature monocytes. The cells were stained with 1 µl of the fluorescently labeled antibody (or the appropriate control immunoglobulin) for 1 h on ice. Next, they were washed with ice-cold phosphate-buffered saline (PBS) and suspended in 0.5 ml PBS supplemented with 0.1 % bovine serum albumin (Sigma, St Louis, MO, USA) prior to analysis on FACS Calibur flow cytometer (Becton–Dickinson, San Jose, CA, USA). The acquisition parameters were set for an isotype control. Cells were analyzed using a FACSCalibur, and data analysis was performed using the CellQuest Pro software (Becton–Dickinson, San Jose, CA, USA).

### Measurement of mRNA and Protein Levels for RARs

Isolation of total RNA, reverse transcription into cDNA, and real-time PCR reactions were performed as described previously (Gocek et al. 2012), using the CFX Real-time PCR System (Bio-Rad Laboratories Inc., CA, USA). The *RARA*, *RARB,* and *RARG* primers were obtained from RealTimePrimers.com (Real Time Primers, LLC, PA, USA), and *GAPDH* primers were as published (Baurska et al. 2011). Quantification of gene expression was analyzed with the ΔΔCq method using *GAPDH* as the endogenous control. Primer efficiencies were measured in all cell lines using a real-time PCR reaction based on the slope of the standard curve. The results were normalized to primer efficiencies to compare gene expression in different cell lines. Real-time PCR assays were performed at least in triplicate.

### Western Blotting

The cytosolic and nuclear lysates were obtained using NE-PER Nuclear and Cytoplasmic Extraction Reagent (Thermo Fisher Scientific Inc., Worcester, MA, USA) according to the user’s manual. 6 × 10^6^ cells per sample were washed with PBS and lysed in 200 μl of cytoplasmic extraction buffer, and centrifuged, and the remaining pellets were lysed in 100 μl of nuclear extraction buffer. The buffers contained a cocktail of protease inhibitors (BioTools, Inc., Jupiter, FL, USA). The lysates obtained were denatured by adding five times sample buffer and boiling for 5 min. For Western blotting, 25 μl of each lysate were separated on 10 % SDS-PAGE gels and transferred to PVDF membranes. The membranes were then dried, and incubated sequentially with primary antibody (3 h) and a horseradish peroxidase-conjugated secondary antibody (1 h) at room temperature. The protein bands were visualized by chemiluminescence (Santa Cruz).

### Retinoid Receptor Transactivation Assays

For transactivation studies, we used CV-1 kidney fibroblast cells and LNCaP prostate adenocarcinoma cells that can be readily and reproducibly transfected with plasmids and reporters. Transactivation assays using CV-1 cells were undertaken essentially as described previously (Nagpal et al. 1995). Briefly, CV-1 cells were transiently transfected with a plasmid-containing ERE-tk-Luc promoter-reporter construct using Lipofectamine as described in the manufacturer’s protocol (Fisher Scientific, Loughborough, UK). ERE-tk-Luc transcript comprises a luciferase reporter under control of an estrogen receptor response element. The cells were also transiently transfected with expression vector encoding a fusion protein containing the ligand-binding domain of RARα, RARβ, or RARγ fused to an estrogen receptor DNA-binding domain. Twenty-four hours after transfection, the cells were treated with retinoids under test for 16 h in culture medium-containing 2.5 % charcoal-treated FBS and lysed for determination of luciferase and β-galactosidase activities. Luciferase activity was measured using the Dual-Luciferase Reporter 100 Assay System (Promega, Southampton, UK), and β-galactosidase activity was determined by colorimetric assay. The reporter activity was normalized against β-galactosidase activity. The transactivation data are presented as the percentage ± SEM of the maximal response produced by 1 μM ATRA. The prostate adenocarcinoma cell line LNCaP was transiently transfected with a pSG5-RARα1 or pSG5-RARγ1 mammalian expression vector together with a pT109-DRG5-luc luciferase reporter under the control of a DR5G RARE (constructed by Dr. Kevin Petrie and Arthur Zelent, Institute of Cancer Research, Sutton, UK). Cells were transfected using Lipofectamine according to the manufacturer’s instructions and treated and assayed as above.

### Statistical Analyses

All assays were performed in triplicate. Results were statistically analyzed by a one-way ANOVA and Student’s *t* test to determine the significance of differences in values obtained from control and treated groups using the Sigmastat™ software package (Systat Software Inc. London). Difference in statistical significance was set at *p* ≤ 0.05. Concentration response curves were fitted to the Hill equation and ED_50_s estimated using the SigmaPlot™ software package (version 10 containing the “pharmacology” module, Systat Software Inc. London).

## Results

### Pharmacological Properties of the Synthetic RAR Isoform-Specific Retinoids

The RAR-selective compounds are highly specific agonists and antagonists as reported elsewhere (Hammond et al. 2002; Hughes et al. 2006; Nagpal et al. 1995). The agonists stimulate transactivation in CV-1 cells transfected with an appropriate RAR expression vector and reporter construct. The antagonists failed to do so for RAR transfected CV-1 cells, and instead, blocked ATRA- and TTNPB-stimulated RAR transactivation in a dose responsive fashion. The binding affinities of compounds at individual isoforms of RAR and RXR are shown in Table 1. The receptor selective α agonist AGN195183 binds with a high affinity to RARα (ED_50_ 20.1 nM). The pan-antagonist AGN194310 and receptor selective α antagonist AGN196996 and γ antagonist AGN205728 bind with high affinities to all RARs (ED_50_ 4.3, 5, and 2 nM), RARα (ED_50_ 3.9 nM), and RARγ (ED_50_ 3 nM), respectively.

**Table 1:** Binding affinities (ED_50_ in nM) of synthetic retinoids against different RAR isoforms

Retinoids	RARα	RARβ	RARγ	Classification
RAR agonists				
AGN195183	20.1	>5000	>5000	RARα
RAR antagonists				
AGN194310	4.3	5	2	RARαβγ
AGN196996	3.9	4036	>10,000	RARα
AGN205728	2400	4248	3	RARγ

Nuclear extracts were prepared from baculovirus-infected Sf21 insect cells engineered to express either human RARα, β, or γ, or RXRα, β, or γ as described (Allegretto et al. 1993; Nagpal et al. 1995). The equilibrium-binding affinities of each retinoid analog (ED_50_ in nM) were estimated by the abilities of non-labelled synthetic retinoids to compete with the binding of [^3^H]-ATRA to a RAR isotype as described (Heyman et al. 1992). Affinities against RXRs were >10,000 nM

### Agonizing RARα Drives Differentiation of NB-4 and KG1 Cells

The human promyeloid cell line HL60 differentiates towards neutrophils in response to ATRA (Breitman et al. 1980). Previously, we used agonists and antagonists of RAR isoforms and retinoid X receptor and a HL60 sub-line that expresses a dominant-negative RARα to show that ATRA-provoked differentiation of HL60 cells requires activation of RARα which forms heterodimers with retinoid X receptor (Hughes et al. 2006).

**Fig. 1:**
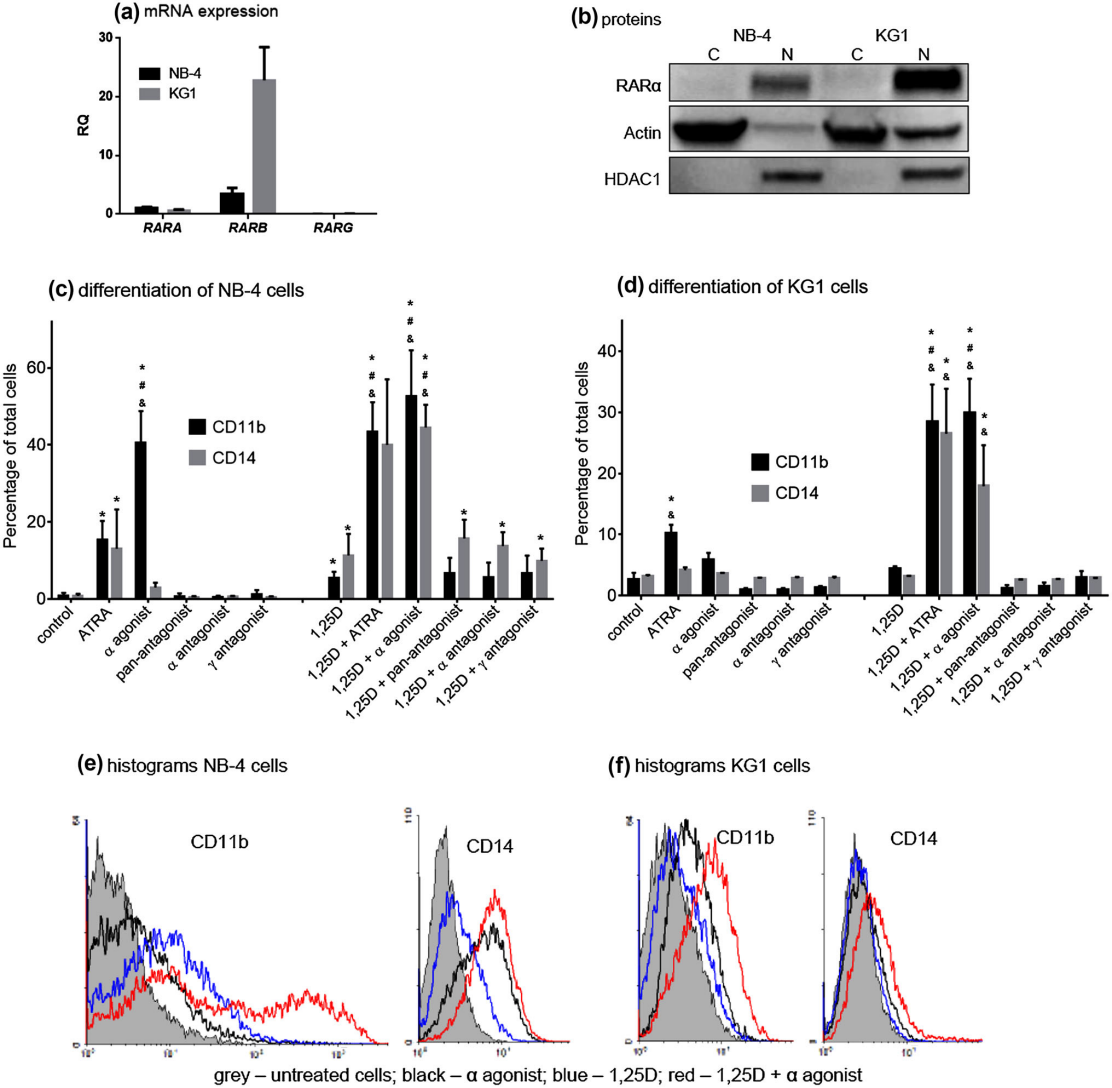
Agonism of RARα is sufficient to drive differentiation of NB-4 and KG1 cells. The expression of RAR isoforms was measured in NB-4 and KG1 cells as to mRNA (**a**) and protein (**b**) levels. The expressions *RARA*, *RARB,* and *RARG* were examined in NB-4 and KG1 cells by Real-time PCR and relative to *GAPDH* expression levels. The expression level obtained for *RARA* in NB-4 cells was calculated as 1. The *bar charts* show mean values (±SD) of the relative quantity (RQ). The levels of RARα protein were determined in NB-4 and KG1 cells by Western blots. The cytosolic (C) and nuclear (N) extracts were separated by SDS-PAGE, transferred to PVDF membranes, and the proteins were revealed using anti-RARα, anti-actin, and anti-HDAC1 antibodies. NB-4 (**c**) and KG1 (**d**) cells were exposed to 1 µM ATRA or to 100 nM synthetic retinoids with or without 10 nM 1,25D for 96 h. The synthetic retinoids were the α agonist AGN195183, pan-antagonist AGN194310, α antagonist AGN196996, and the γ antagonist AGN205728. Cell surface expression of the CD11b and CD14 differentiation markers was detected by flow cytometry to enumerate cells that had differentiated towards neutrophils (CD11b^+ve^CD14^−ve^) and monocytes (CD11b^+ve^CD14^+ve^). The *bar charts* show the mean ± SEM of the values obtained from triplicate cultures. *Values that are significantly higher than control sample; ^#^values that are significantly higher than ATRA-treated sample and ^&^values that are significantly higher than 1,25D-treated sample. Representative histograms are presented for NB-4 (**e**) and KG1 (**f**) cells. The *grey area* represents the expression of a given cell surface marker in untreated cells, the *black line* in α agonist-treated cells, the *blue line* in 1,25D-treated cells, and the *red line* in cells treated with combination of both agents

The NB-4 cell line harbors one copy of the wild-type *RARA* gene and one copy of the *PML*-*RARA* fusion gene, which encodes the fusion protein PML-RARα (Lanotte et al. 1991). KG1 cells express the fusion gene *FGFR1OP2*-*FGFR1* encoding the fusion protein FOP2-FGFR1 which activates the STAT signaling pathways (Gu et al. 2006). The expression levels of *RARA*, *RARB,* and *RARG* genes in these cells were measured, relative to *GAPDH* expression levels, using Real-time PCR. Figure 1a shows that both cell lines express high levels of *RARB*, moderate levels of *RARA*, while *RARG* mRNA is almost undetectable. The presence of respective proteins in cytoplasmic and nuclear fractions of lysates, prepared from equal numbers of NB-4 and KG1 cells, was investigated by Western blotting (Fig. 1b). Comparison of Fig. 1a and b reveals that the levels of protein and mRNAs do not match. The RARα isoform was readily detected in lysates and the antibodies against RARβ and RARγ did not give a detectable signal. RARα was detected only in lysates of the nuclei of both cell lines with KG1 cells expressing RARα at a higher level than NB-4 cells, when normalized to the level of histone deacetylase 1 (HDAC1), which also confirmed the purity of nuclear lysates.

Figure 1c shows that NB-4 cells differentiated towards monocytes (CD11b^+ve^/CD14^+ve^) to a small extent when treated with a high dose (1 μM) of ATRA (~10 % CD11b^+ve^/CD14^+ve^ cells) and a low dose (10 nM) of 1,25D (~6 % CD11b^+ve^/CD14^+ve^ cells). The combined use of 10 nM 1,25D and 1 μM ATRA increased the level of monocyte differentiation to ~36 %. Figure 1c shows that 100 nM of the α agonist AGN195183 effectively induced neutrophil differentiation of NB-4 cells to a level of 40 % CD11b^+ve^/CD14^−ve^ cells. AGN195183 was also more effective than ATRA when used in combination with 10 nM 1,25D to induce monocyte differentiation. 100 nM of AGN195183 was sufficient, as compared with 1 μM ATRA, to give rise to 40 % CD11b^+ve^/CD14^+ve^ cells. Pan, α, and γ antagonists had no effect on NB-4 cells when used alone, and the antagonists did not have an appreciable effect when used with 10 nM 1,25D as compared with the use of 1,25D alone.

Figure 1d shows that treatment of KG1 cells with ATRA and the α agonist AGN195183 led to a low level of neutrophil differentiation of between 5 and 10 % CD11b^+ve^/CD14^−ve^ cells. Both ATRA and AGN195183 when used in combination with 10 nM 1,25D resulted in a significant level of differentiation towards monocytes. Percentages of CD11b^+ve^/CD14^+ve^ cells were 26 and 18 %, respectively. Again, the α agonist was observed to be more potent than ATRA, as this agent was used at 100 nM compared to 1 μM ATRA. The pan, α, and γ antagonists had no effect on KG1 cells when used alone and together with 1,25D. Representative histograms which show the levels of differentiation induced by the α agonist, 1,25D or both agents are presented in Fig. 1e for NB-4 cells and in Fig. 1f for KG1 cells.

### Antagonism of RARs Increases the Production of Myeloid Cells by CD34^+ve^huHSC-Initiated Cultures

The phenotype of the CD34 column purified cells as to surface markers associated with hematopoietic stem and progenitor cells was 95.3 ± 1.7 CD133^+ve^, 98.1 ± 0.6 CD34^+ve^, 84.7 ± 4.7 CD117^+ve^, 90.1 ± 3.0 CD38^+ve^, and 91.1 ± 3.0 HLA-DR^+ve^ (*n* = 15 donors). The levels of contamination by myeloid- (CD14, CD15, CD11b and CD64), erythroid- (glycophorin A), T-cell- (CD3, CD5, CD7, CD8 and CD27), and B-cell lineage- (CD19) associated cells were 0.9 ± 0.1, 0.8 ± 0.1, 1.0 ± 0.1, and 1.6 ± 0.5, respectively.

CD34^+ve^huHSC spontaneously and progressively mature giving rise largely to mature myeloid cells when cultured in a various growth media, sources of serum, and often SCF and IL-3. As such, CD34^+ve^HSC cannot be reliably expanded in culture. To obtain differentiating myeloid cells from CD34^+ve^huHSC in numbers sufficient for biochemical studies, we have routinely cultured these cells in a low amount of human recombinant IL-3 ± G-CSF (Mountford et al. 1994). From past studies 10 % FBS, 100 ng/ml SCF, 5 ng/ml IL-3, and 30 ng/ml G-CSF give the best yield of mature myeloid cells from CD34^+ve^huHSC. We used these conditions as a benchmark to the optimal generation of myeloid cells.

**Fig. 2:**
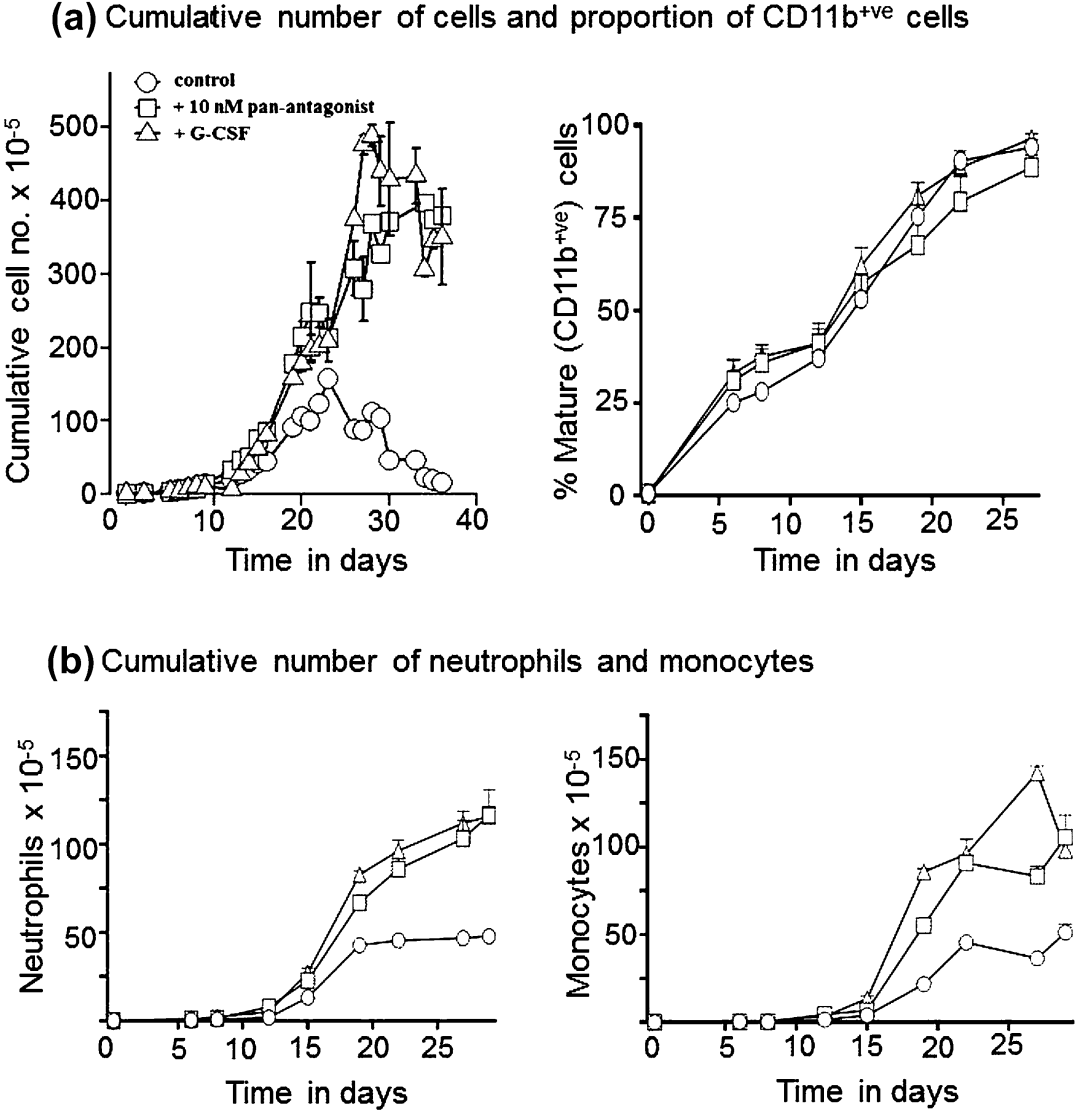
Antagonism of RARs increases neutrophil production in cultures of human haematopoietic stem cells. Purified human haematopoietic stem cells (CD133^+ve^/CD34^+ve^) were cultured from a starting density of 2.5 × 10^5^ cells/ml in RPMI1640 medium with 10 % FBS, 100 ng/ml SCF, and 20 ng/ml IL-3, and these conditions together with 10 nM of the pan-RAR antagonist AGN194310. Cells were cultured in 10 % FBS, 100 ng/ml SCF, 5 ng/ml IL-3, and 30 ng/ml G-CSF to promote the optimal production of neutrophils and monocytes. Cultures were fed with fresh medium plus agents as required and expanded when the cell density reached 1 × 10^6^/ml. **a** Shows the cumulative number of cells and the fraction of mature cells as measured by the FACS analysis of surface expression of CD11b (neutrophils and monocytes). **b** Shows the cumulative number of neutrophils (CD11b^+ve^/CD65^+ve^) and monocytes (CD11b^+ve^/CD14^+ve^). Data are mean ± SEM of the values obtained from triplicate cultures

Control cultures (FBS, SCF, and IL-3) of CD34^+ve^HSC and cultures supplemented with either the pan-RAR antagonist AGN194310 or G-CSF progressively differentiated giving rise mostly to neutrophils and monocytes. During the lifespan of the cultures, the percentage of erythroid cells (glycophorin A) varied between 5 and 8 %, megakaryocyte lineage cells (CD61) were between 7 and 9 %, and B lymphocytes (CD19/CD20) and T lymphocytes/NK cells (CD2/CD3/CD7/CD56) were <1 %. In control cultures, cell production peaked at day 20 with around 1 × 10^7^ cells generated, and mature neutrophils and monocytes were present in almost equal proportions (Fig. 2b). After day 20, cell production declined rapidly and the cultures had started to expire (cell density <0.5 × 10^5^/ml) by day 30. The addition of 10 nM of the pan-antagonist AGN194310 led to a substantial and sustained increase in cell production, as revealed by the cumulative cell number. By day 20, around fourfold more cells had been produced in the antagonist-treated cultures than in control cultures, and the antagonist-treated cultures were maintained for as long as 55 days. An almost identical kinetic was observed for cultures supplemented with 100 nM AGN194310 (data not shown). Increased cell production by the antagonist-treated cultures up to day 20 was not attributable to a higher level of dead/apoptotic cells in control cultures, as few dead/apoptotic cells were present in both culture conditions, as observed by phase contact microscopy when counting cells and cells gated out in FACS analyses. As seen for control cultures, similar numbers of mature neutrophils and monocytes were produced in the antagonist-treated cultures (Fig. 2). The kinetic of increased production of total and myeloid cells by cultures treated with the pan-antagonist AGN194310 was not significantly different from the kinetic observed for cultures supplemented with 30 ng/ml G-CSF (Fig. 2a, b). G-CSF-supplemented cultures were maintained for as long as 55 days and also had produced fourfold more cells by day 30. We examined the extent to which cultures differentiated at similar or different rates by measuring the appearance of CD11b^+ve^ cells (neutrophils and monocytes) and disappearance of CD33^+ve^ immature myeloid cells. As to all culture conditions, CD11b^+ve^-differentiated myeloid cells appeared at a similar rate (Fig. 2a), and immature myeloid cells declined at the same rate (data not shown).

### Short-Term Expansion of CD34^+ve^huHSC and Progenitors Underlies Antagonist-Driven Increased Myeloid Cell Production

The RAR antagonist AGN194310 did not slow down myeloid cell differentiation, and therefore, we examined whether enhanced expansion of CD34^+ve^huHSC and progenitors might underlie the increased production of myeloid cells and extended lifespan of cultures. In a series of experiments, various medium conditions were compared to optimize viability of the purified CD34^+ve^huHSC at the start of experiments. We examined the use of RPMI-1640 versus various hematopoietic growth media, different sera (FBS, horse and human), serum-free medium, SCF ± IL-3, and culturing the cells in normal oxygen levels (20 %), and at hypoxic conditions (1 %). Cell viability measurements and visual inspection of cultures at early time points revealed improved starting cell viability when CD34^+ve^huHSC cells were cultured in the hematopoietic growth medium HPGM^TM^ supplemented with 10 % human serum and in normal oxygen levels. The addition of 100 ng/ml SCF was sufficient to ensure high starting cell viability. In all the above experiments, the levels of expansion of lineage^−ve^/CD133^+ve^/CD34^+ve^huHSC varied five- to eightfold by ~day 30.

**Fig. 3:**
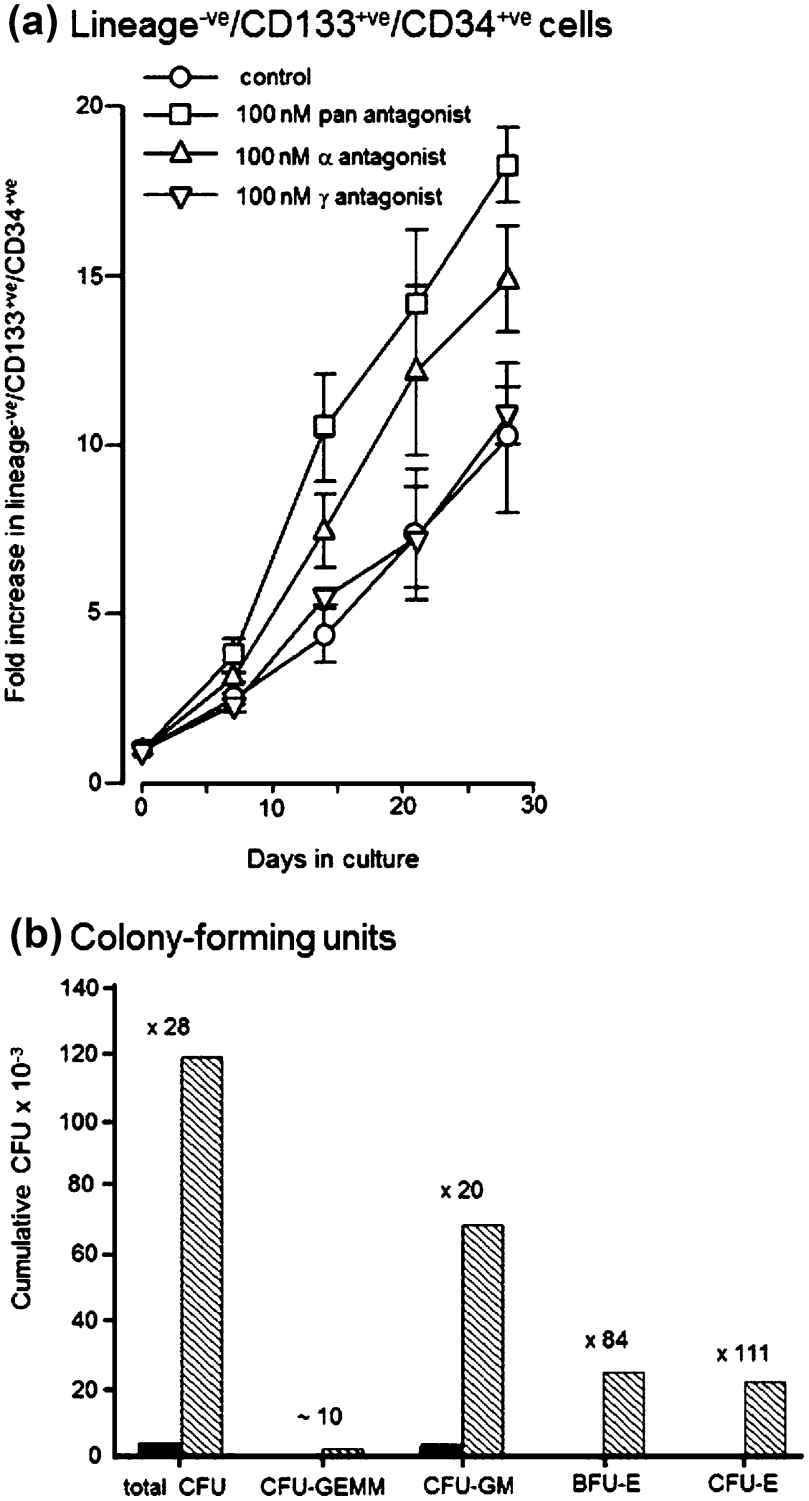
Antagonising RARα enhances ex vivo expansion of human hematopoietic stem cells. **a** Purified lineage^−ve^/CD133^+ve^/CD34^+ve^cells were cultured in HPGM supplemented with 10 % human serum and 100 ng/ml SCF and in the presence of 100 nM of either a pan or α or γ RAR antagonist. Lineage^−ve^/CD133^+ve^/CD34^+ve^cells were measured by the multi-color FACS analysis. The data shown are from seven separate experiments using seven different donors. Data are mean ± SEM. **b** At 2 weeks, cells harvested from cultures of control cells (*shaded bars*), and cells grown in the presence of the pan-RAR antagonist (*cross-hatched bars*) and absence of antagonist (*solid bars*) were plated into a standard methylcellulose assay to determine numbers of various colony-forming cells. The numbers on the *graph* show the fold level of expansion of colony-forming units as compared to control cultures. *CFU* colony-forming unit, *CFU-GEMM* granulocyte/erythroid/megakaryocyte/macrophage colony-forming unit, *CFU-GM* granulocyte/macrophage colony-forming unit, *BFU-E* burst-forming unit-erythroid, *CFU-E* colony-forming unit-erythroid

Figure 3a shows an ~eightfold level of expansion of lineage^−ve^/CD133^+ve^/CD34^+ve^huHSC at day 30 when these cells were cultured in HPGM^TM^ supplemented with 10 % human serum and 100 ng/ml SCF. The addition of the pan-antagonist AGN194310, at 100 nM, led to an increase in the rate of expansion of lineage^−ve^/CD133^+ve^/CD34^+ve^huHSC and the level of expansion of these cells, was increased to ~18-fold at day 30 (mean value for seven donors). Antagonizing RARα was sufficient to enhance expansion of lineage^−ve^/CD133^+ve^/CD34^+ve^ huHSC and antagonizing RARγ did not improve cell expansion (Fig. 3a). In experiments using a further eight different donors, we used the pan-antagonist and the α antagonist. For all 15 donors, antagonizing either all RARs or RARα enhanced expansion of lineage^−ve^/CD133^+ve^/CD34^+ve^ cells to the extent of around 20-fold, as compared to an 8–10-fold expansion seen in control cultures. Enhanced expansion occurred when cells from some donors were cultured in both flasks and wells.

For CD34^+ve^huHSCs cultured in wells for 2 weeks in the presence and absence of the pan-RAR antagonist AGN194310 (at 100 nM), we examined the presence of week-five CAFC, by plating onto MS5 feeder cells, and whether established week-five CAFC cultures were able to produce CFUs, to measure the presence of LTC-IC. CAFC and LTC-IC were only present in pan-RAR-antagonized wells (two donors). For 2 donors and at 2 weeks, we also examined the presence of CFUs in cultures grown in the presence and absence of the pan-antagonist AGN194310 (at 100 nM). As to culture of cells with the pan-antagonist AGN194310 vs control cultures, CFU-GEMM were 3 % of the total colony-forming units versus not detected, CFU-GM were 56 vs 93 %, and BFU-E together with CFU-E were 41 vs 7 %. The presence of the antagonist led to increases in the total number of CFUs, CFU-GEMM, and CFU-GM of between 10- and 30-fold. In control cultures, BFU-E/CFU-Es were at a very low level (7 % of total colonies) and the presence of the antagonist led to a high value for the expansion of these CFUs of 80- to 110-fold (Fig. 3b). This increase in BFU-E/CFU-E might be related to antagonism of the effect of ATRA that is present in FBS, as ATRA can specify a granulocyte fate (Tocci et al. 1996). Despite the sustained presence of CFUs, including maintenance of CFU-GEMM, in antagonist-treated cultures, these cultures gradually differentiated to produce neutrophils and macrophages, as shown in Fig. 2.

### Selective Transactivation of RARα, RARβ, and RARγ by ATRA Is Concentration Related

The RARγ antagonist did not have an effect on the expansion of lineage^−ve^/CD133^+ve^/CD34^+ve^huHSC (Fig. 3), and the kinetics of the production of total cells, neutrophils, and monocytes observed for RARγ antagonist-treated cultures were identical to those shown for the control culture in Fig. 2. By day 20, around fourfold more cells (largely myeloid) had been produced in the α antagonist-treated cultures than in the γ antagonist-treated and control cultures.

**Fig. 4:**
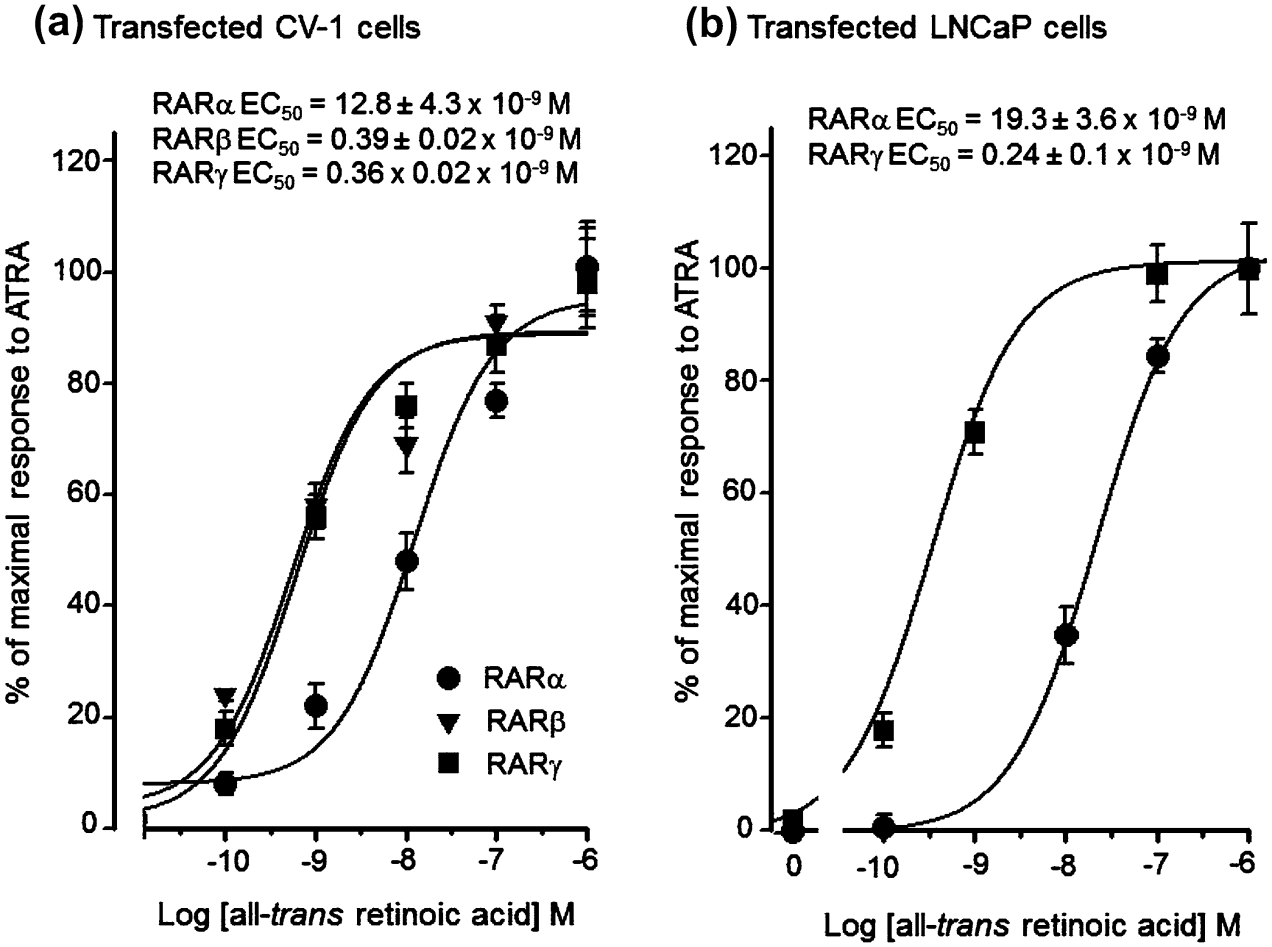
ATRA stimulates transactivation via RARγ and RARβ at sub-nanomolar concentrations, whilst much higher concentrations are required for RARα-mediated transactivation. The effect of increasing concentrations of ATRA on RARα-, RARβ-, and RARγ-mediated transactivation in **a** CV-1 cells and **b** serum-free-adapted LNCaP cells. Cells were transfected as described in the experimental section and were treated with 10^−10^ to 10^−6^ M ATRA for 24 h. Results shown are from a typical experiment performed in triplicate and are expressed as RLU (arbitrary units) ± SEM. To correct for differences in transfection efficiency between samples values were normalized with respect to β-galactosidase activity

A closed helix 12 conformation in RARγ, and RARβ has been reported to block binding of corepressors, and these RARs may activate expression of target genes in the absence of ligand (Farboud et al. 2003; Hauksdottir et al. 2003). We examined the levels of ATRA required to agonize RARs by transfecting CV-1 and LNCaP cells with an expression vector for each of the RAR isoforms together with an RAR reporter construct and treating these cells with ATRA. At concentrations below 10^−9^ M, preferential transactivation of RARβ and RARγ was observed (EC_50_’s = 0.39 × 10^−9^ and 0.36 × 10^−9^ M, respectively), with maximal activation occurring at ~5 × 10^−9^ M ATRA. ATRA had a minimal effect on transactivation via RARα at concentrations below 10^−9^ M (EC_50_ = 12 × 10^−9^ M) and a maximal effect was not observed until the ATRA concentration approached 10^−7^ M (Fig. 4a). Figure 4b shows LNCaP cells transfected with an expression vector for RARγ and reporter plasmid that ATRA also stimulated transactivation at sub-nanomolar concentrations (EC_50_ = 2.4 ± 1.0 × 10^−10^ M). Much higher concentrations of ATRA were required to produce significant transactivation of RARα for LNCaP cells transfected with this isoform (EC_50_ = 1.9 + 0.4 × 10^−8^ M) (Fig. 4b). These findings reveal that a tissue concentration of ATRA of ~10^−9^ M and below will selectively activate RARγ, and a much higher concentration (≫10^−9^ M) is required to maximally activate RARα.

## Discussion

Previous studies have shown that agonizing RARα is sufficient to drive growth arrest and neutrophil differentiation of the promyeloid cell line HL60. Similarly, the RARα agonist was effective in driving neutrophil differentiation of NB-4 cells, and like ATRA, it enhanced monocyte differentiation of NB-4 and KG1 cells when used in combination with a low dose of 1,25D. In regard to the later, the RARα agonist was more potent than ATRA, as was used at 100 nM vs 1 μM ATRA. These findings are in keeping with activation of RARα which promotes myeloid cell differentiation.

Antagonizing RARs in cultures of CD34^+ve^huHSCs increased the generation of mature neutrophils and monocytes and prolonged the lifespan of the culture. We did not see a slowing down of differentiation, and the increased myeloid cell production was due to the enhancement of the short-term expansion of CD34^+ve^huHSC and progenitor cells. Abrogating RARα activity was sufficient to enhance expansion of CD34^+ve^huHSC and increase myeloid cell production. The improved level of expansion of CD34^+ve^huHSC may not be sufficient to be of benefit to rapidly expanding these cells in the case of an inadequate harvest for transplantation. However, RAR antagonist-provoked increased neutrophil production may be of benefit to patients with neutropenia if the effect can be seen in vivo.

Early work by Purton et al. (1999) highlighted the importance of retinoids to the differentiation of primitive hematopoietic cells. These workers showed that ATRA delays the differentiation of precursor cells, enhances the terminal maturation of progenitors that are committed to granulocyte/monocyte differentiation (Purton et al. 1999), and enhances the long-term repopulating activity of cultured HSCs (Purton et al. 2000). Walkley et al. (2002) and Chee et al. (2013) administered the pan-antagonist AGN194310 to mice and observed increases in myeloid progenitor cells and mature myeloid cells. As such, findings from these mouse studies and the CD34^+ve^huHSC culture experiments are in agreement. An AGN194310-provoked expansion of granulopoiesis was not seen in knockout mice lacking the receptor for G-CSF (G-CSFR) and Chee et al. (2013) hypothesized that G-CSFR signaling and RARs interact to regulate myeloid cell differentiation. In this regard, similar enhancement of myelopoiesis was seen in cultures of CD34^+ve^huHSC supplemented with either AGN194310 or G-CSF: the pan-antagonist appears to mimic the presence of G-GSF. The antagonist-provoked expansion of myelopoiesis may not be intrinsic to mouse and human HSC, and Chee and co-workers (2013) have concluded that the increase in myeloid cells in mice treated with AGN194310 was due to an increased production of G-CSF. This is a possibility for AGN19430-treated cultures of CD34^+ve^huHSC, as macrophages appear early, increase in number, and produce G-CSF (Chang et al. 2015; Demetri and Griffin 1991; Hareng and Hartung 2002). Even so, pathways that are both GSF-independent and RAR-independent exist to ensure granulopoiesis, as neutrophils are produced in mice doubly null for G-CSF and RARα or RARγ (Chee et al. 2013).

It was surprising to observe that the RARγ antagonist did not significantly affect cultures of CD34^+ve^huHSC, as Purton and co-workers have reported that RARγ-knockout mice have a reduced number of HSC. These workers also investigated the repopulating capacity of lineage^−ve^c-kit^+ve^Sca1^+ve^ (LSK^+^) cells from wild-type, RARγ^+/+^, RARα^−/−^, and RARγ^−/−^-mutant mice after culturing these cells for 14 days with ATRA. The findings from these experiments led to the conclusion that RARγ mediates the balance between HSC self-renewal and differentiation (Purton et al. 2006). However, the findings from studies of the influence of RARγ on cultures of CD34^+ve^huHSC and mouse LSK^+^ cannot be compared directly for two reasons. The absence of receptor (null mice) and use of ATRA, in the case of the mouse LSK^+^ experiments, is not the same as the presence of antagonized or otherwise RARγ as to the CD34^+ve^huHSC experiments. In addition, mouse LSK^+^ cells and CD34^+ve^huHSC are different in the extent of their cellular heterogeneity. In particular, CD34^+ve^huHSC are a less pure population of “true” stem cells which may have precluded seeing an effect on cells that are repopulating.

The role of RARγ is confounded by reports that this receptor does not repress and functions as an agonist when ligand is absent/undetectable (Farboud et al. 2003; Hauksdottir et al. 2003). We examined the possibility that RARγ is activated by an exceedingly low level of ATRA. Indeed, reporter assays revealed that ATRA activates RARγ at sub-nanomolar concentrations (10^−10^ M): a much higher concentration is required to activate RARα (10^−8^ M). The importance of this preferential activation is the overall tissue level of ATRA is low, as measured by high-performance liquid chromatography-mass spectrometry (Bleul et al. 2015), and RARs will be differentially activated, as the local concentration varies. In particular, the levels of ATRA in embryonic tissues are tightly controlled, gradients affect differential gene expression, and correct development (Rhinn and Dollé 2012), and boundaries are controlled by the ATRA-metabolizing enzyme retinaldehyde reductase DHRS3 (Billings et al. 2013). In vitro, stem cell differentiation can be regulated by controlling the ATRA gradient in a 3D scaffold (Tzezana et al. 2012). A striking example of the influence of local increase in the level of ATRA is that production by dendritic cells in the gut imprints the phenotype and gut homing tropism of effector T cells (Iwata et al. 2004).

In this study, a pharmacological amount of ATRA (1 μM) was used to differentiate NB-4 and KG1 cells, which is in keeping with activation of RARα to favor differentiation. Nanomolar ATRA should just lead to transactivation of RARγ, and the physiological importance of selectively activating RARγ has been revealed by treating zebra fish embryos with a low amount (10 nM) of an RARγ agonist (AGN205327). Stem cell populations were maintained at the expense of the development of bones and neural ganglia from cranial neural crest stem cells and pectoral and caudal fins from mesodermal stem cells. Appropriate development was restored by agonist washout or reversal with the RARγ antagonist. Hence, RARγ remaining in its non-ligand bound state or at a low level of activation is important to whether the above stem cell populations make a decision to adopt a fate and/or differentiate (Wai et al. 2015).

In summary, the findings reported here and by other workers support the notion that the balance of expression of RARα and RARγ and activities of these receptors, which can be governed by the local availability of ATRA, are important to HSCs retaining this status or embarking on myeloid cell differentiation. RARs and G-CSF appear to cooperate in their action which is of particular interest as growth factors, that include G-CSF, GM-CSF, M-CSF, erythropoietin, and Flt3 ligand, can instruct cell-fate decisions (reviewed in Brown et al. 2015; Grover et al. 2014; Metcalf 1991; Metcalf and Burgess 1982; Mossadegh-Keller et al. 2013; Rieger et al. 2009; Tsapogas et al. 2014). An understanding of the cooperative actions of RARs and G-CSF may well provide a better resolve to how HSCs govern their status.
